# Resistance and virulence distribution in enterococci isolated from broilers reared in two farming systems

**DOI:** 10.1186/s13620-021-00201-6

**Published:** 2021-08-20

**Authors:** Teresa Semedo-Lemsaddek, João Bettencourt Cota, Tânia Ribeiro, Amélia Pimentel, Luís Tavares, Fernando Bernando, Manuela Oliveira

**Affiliations:** CIISA–Centre for Interdisciplinary Research in Animal Health, Faculty of Veterinary Medicine, University of Lisbon, Avenida da Universidade Técnica, 1300-477 Lisbon, Portugal

**Keywords:** Antimicrobial resistance, *Enterococcus*, Broilers, Resistance genes, Virulence genes, Farming system

## Abstract

**Background:**

The impact of enterococci in human health has been growing for the last decades, mainly due to their resistance to several antimicrobial agents. Human consumption of contaminated meat, especially poultry, has been identified as a possible route of transmission. The aim of the present study was to evaluate and compare the antimicrobial resistance profiles and virulence genes of enterococci isolated from Portuguese conventional and free-range broiler farms.

**Results:**

Antibiotic susceptibility testing showed high frequencies of resistance to tetracycline in both farming systems. Resistance to erythromycin and gentamicin were detected in about half of the isolates. Resistance to penicillin was the less frequently observed and no vancomycin resistant isolates were identified. The majority of the enterococcal isolates, from either farming systems, were resistant to more than one antibiotic, and no statistical associations were found, except for penicillin resistance which associated with the genetic clusters. No differences were found between farming systems regarding the prevalence of *tet*(M), *erm*(B), *aac (6′)-Ie-aph (2″)-Ia* and *pbp5* genes, nevertheless *pbp5* prevalence was associated with the different genetic clusters. Hemolytic activity was identified in 26.47% of all isolates and gelatinase activity in 50%. The *gel*E gene was identified in the majority of the isolates, whereas *esp* and *agg* genes were rarely detected. The *cyl*A determinant was not detected in any of the isolates.

**Conclusions:**

Overall, results suggest that similar resistance patterns and virulence genes can be found in both farming systems, though enterococci in free-range conditions should be less prone to acquire further resistance genes.

## Background

Enterococci belong to the commensal microbiota found in the intestinal tract of animals and humans [1], but they can also be found in the environment in soil, plants and water [2]. For some years now, enterococci have been regarded as increasingly important human opportunistic pathogens due to their association with severe clinical cases of endocarditis, bacteremia, and urinary tract, central nervous system and nosocomial infections [3, 4].

One of the major concerns regarding these opportunistic pathogens is their frequent antimicrobial resistance (AMR) profile. Enterococci are intrinsically resistant, at variable levels, to commonly used antimicrobial compounds such as β-lactams, cephalosporins and aminoglycosides thus hampering the treatment of enterococcal infections [5]. Similarly, acquired AMR in these bacteria is of great importance. Due to their ability to gain foreign genetic material, including transposons and plasmids, enterococci rapidly became resistant to additional antimicrobial agents such as erythromycin and tetracyclines, shortly after their introduction into clinical practice [6]. Owing to their characteristics, it is believed that enterococci play a pivotal role in the acquisition, conservation and dissemination of AMR genes to other related bacteria [7].

The virulence of enterococci is not exclusively associated with their known AMR traits. The pathogenesis associated with enterococci infections arises from the capability of these bacteria to adhere, invade and to multiply in different environmental conditions [3]. Some of the major virulence factors associated with enterococcal infections are adhesins, such as the enterococcal surface protein (Esp) and the aggregation substance (AS), encoded in the *esp* and *agg* genes respectively, and different enzymes with proteolytic capability (gelatinase or hemolysin) associated with the products of *gel*E and *cyl*A genes, among others [8, 9].

One of the proposed routes of human infection by AMR enterococci is the consumption of contaminated fresh or processed meats [10]. In fact, food-chains started being regarded as possible sources of AMR enterococci since some studies reported the presence of these bacteria in beef, pork or poultry meat [11–14]. Fecal contamination of the carcasses can occur during slaughter, potentially leading to the dissemination of these pathogens to fresh meat, originating from the gastrointestinal tract of apparently healthy animals [8, 10].

Within the scope of food-borne AMR enterococci, poultry meat can represent a greater risk to human health when compared with beef or pork due to the higher incidence of resistance genes found in AMR enterococci detected in retail chickens [12]. Consumer concerns regarding the amounts of antimicrobials used and the rise of AMR levels has steered some conventional (indoor) poultry farms to restructure and change to a free-range or organic production method. Though limited, in the recent years some studies have compared the presence of AMR enterococci from indoor and free range or organic chickens. Results indicate the presence of lower levels of antimicrobial resistance in enterococci of free range or organic production methods when compared with isolates obtained at the indoor systems [15–17]. Similar information concerning resistance levels in enterococci from Portuguese conventional and alternative poultry farms is scarce.

The aim of the present study was to evaluate and compare the profiles of antimicrobial resistance and virulence genes of enterococci obtained from conventional and free-range Portuguese broilers, bringing additional data to the body of knowledge regarding AMR enterococci as well as their possible implications in human and animal health.

## Results

### Microbial diversity

**Fig. 1:**
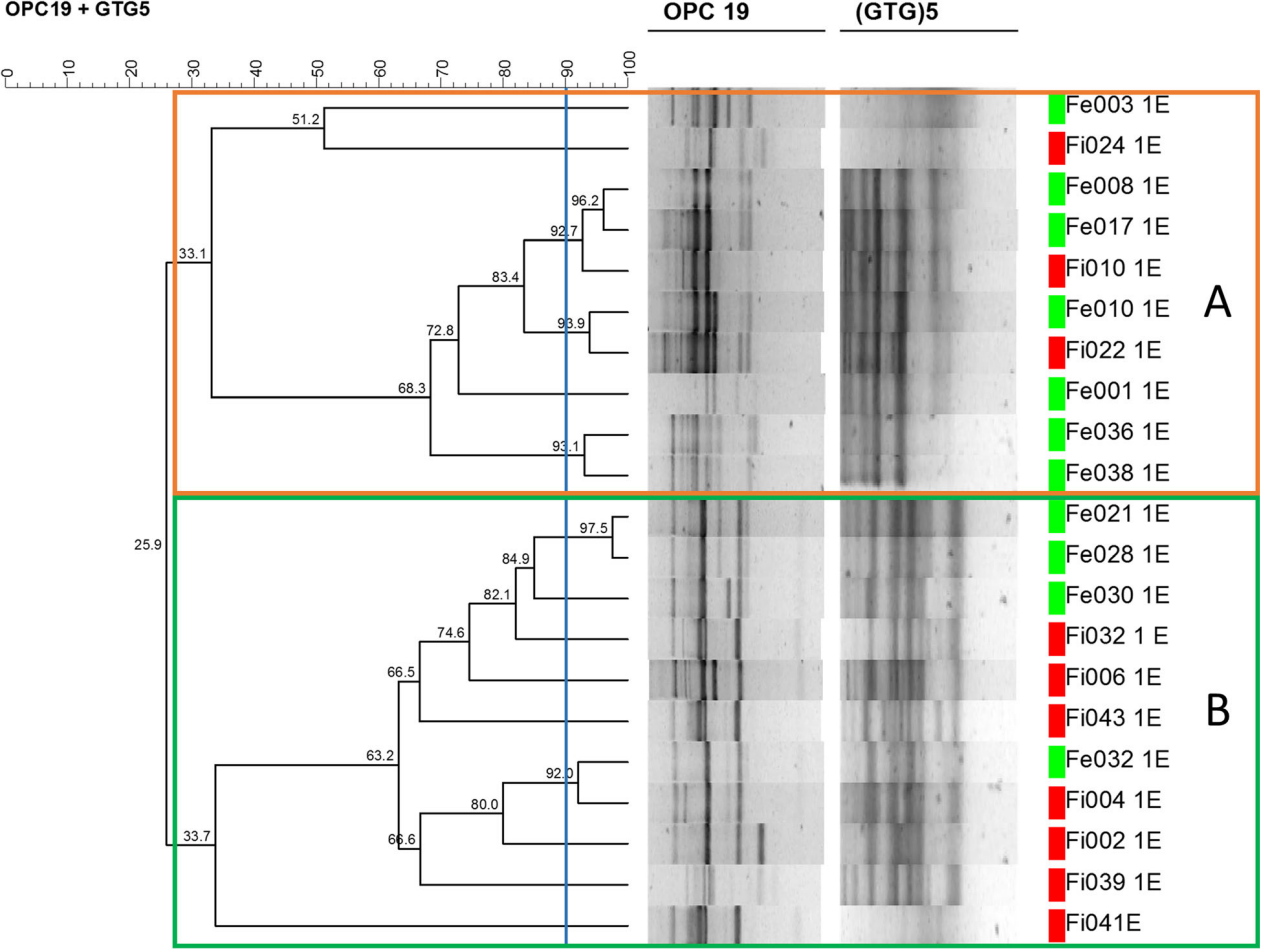
Dendrogram based on genomic patterns of *E. faecium* isolates obtained with primers OPC19 and (GTG)5. Similarity was calculated with Pearson product-moment correlation coefficient, and clustering was performed with the unweighted pair group method with arithmetic mean algorithm (UPGMA). The blue line indicates the reproducibility level

**Fig. 2:**
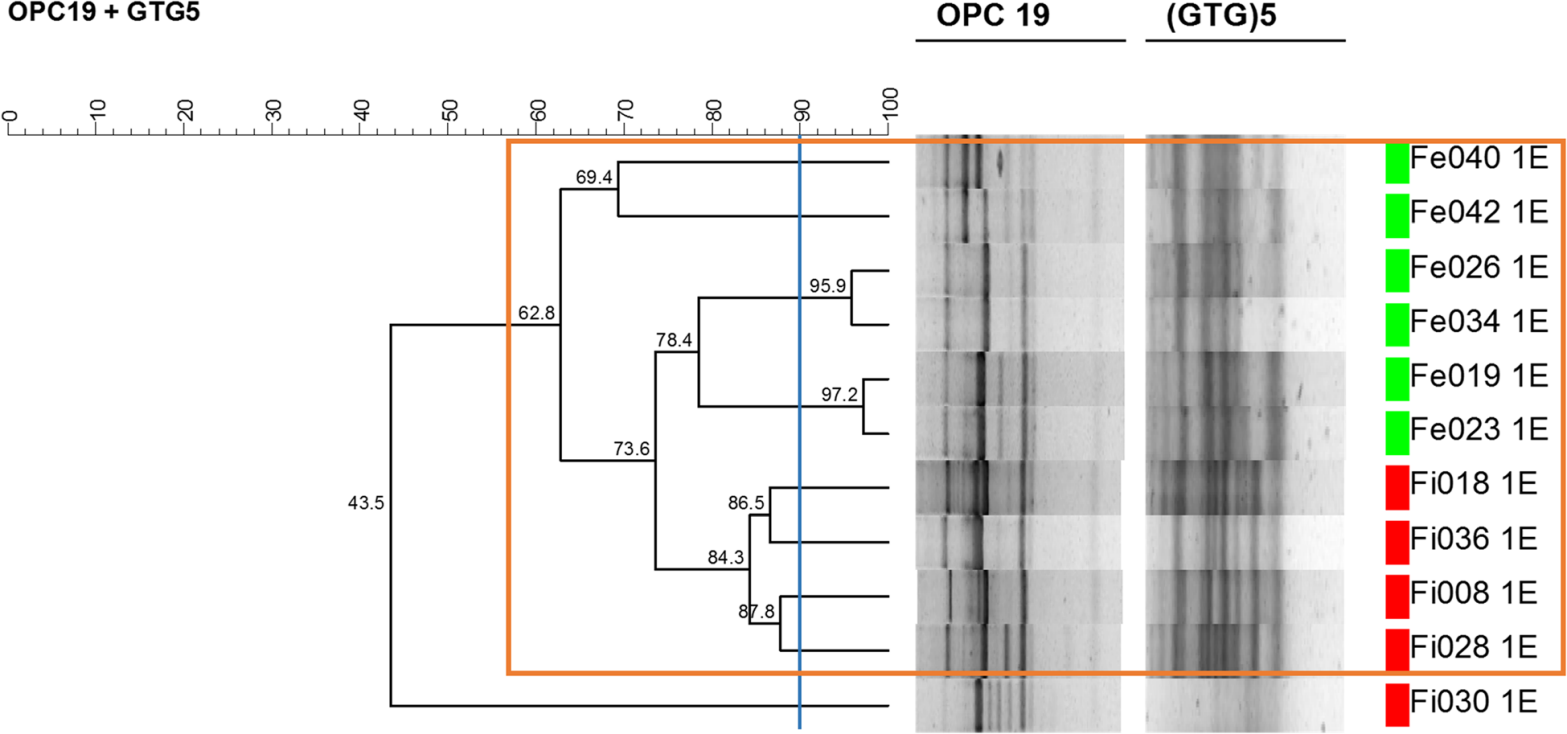
Dendrogram based on genomic patterns of *E. faecalis* isolates obtained with primers OPC19 and (GTG)5. Similarity was calculated with Pearson product-moment correlation coefficient, and clustering was performed with the unweighted pair group method with arithmetic mean algorithm (UPGMA). The blue line indicates the reproducibility level

Of the 34 enterococci isolates obtained from the broiler fecal samples recovered from the two slaughterhouses, 21 were identified as *E. faecium*, 11 as *E. faecalis*, one as *E. gallinarum* and one as *E. durans*. PCR-fingerprinting with primers OPC-19 (*5′-GTTGCCAGCC-3′*) and (GTG)_5_ was performed and followed by clustering analysis of genomic profiles of *E. faecium* and *E. faecalis* isolates. The corresponding dendrograms are depicted in Figs. 1 and 2.

Regarding *E. faecium* isolates, two main clusters were observed - A and B – with a relative similarity level of 30% (Fig. 1), including isolates of both farming system in either cluster. Additionally, some *E. faecium* isolates recovered from different rearing systems had similarity levels above 90%. Cluster A included 10 isolates (orange), while cluster B (green) harbored the remaining 11 isolates.

The cluster analysis of the genomic profiles of *E. faecalis* isolates (Fig. 2) revealed one major cluster (orange), comprising the majority of the studied isolates (10/11), while the remaining isolate did not cluster.

### Antibiotic resistance

Antibiotic resistance profiles of the enterococci isolates used in the present study are displayed in Table 1.

**Table 1:** Antimicrobial resistance and virulence pheno and genotype distribution in conventional and free-range broiler enterococci and in the genomic clusters

		Conventional (***n*** = 18) (%)	Free-range (***n*** = 16) (%)	***P*** value	***E. faecium*** cluster A (***n*** = 10) (%)	***E. faecium*** cluster B (***n*** = 11) (%)	***E. faecalis*** cluster (***n =*** 10) (%)	***p value***
**Antimicrobial resistance**								
Phenotype	TE	14 (77.7%)	14 (87.5%)	N.S.	6 (60%)	10 (90.9%)	9 (90%)	N.S
	E	10 (55.6%)	6 (37.5%)	N.S.	3 (30%)	8 (72.7%)	5 (50%)	N.S.
	CN	9 (50%)	7 (43.8%)	N.S.	4 (40%)	5 (45.5%)	7 (70%)	N.S.
	P	6 (33.3%)	1 (6.25%)	N.S.	6 (60%)	0	1 (10%)	0.002
	VAN	0	0	–	0	0	0	–
Genotype	*tet*(M)	9 (50%)	9 (56.25%)	N.S.	3 (30%)	5 (45.5%)	7 (70%)	N.S.
	*erm*(B)	8 (44.4%)	4 (25%)	N.S.	2 (20%)	5 (45.5%)	4 (40%)	N.S.
	*aac(6′)-Ie-aph(2″)-Ia*	5 (27.7%)	1 (6.25%)	N.S.	1 (10%)	2 (18.2%)	3 (30%)	N.S.
	*pbp5*	6 (33.3%)	1 (6.25%)	N.S.	6 (60%)	0	1 (10%)	0.002
**Virulence**								
Phenotype	Hemolytic activity	6 (33.3%)	3 (18.75%)	N.S.	6 (60%)	1 (9.1%)	0	0.004
	Gelatinase activity	8 (44.4%)	9 (56.25%)	N.S.	1 (10%)	9 (81.8%)	6 (60%)	0.003
Genotype	*cyl*A	0	0	–	0	0	0	–
	*gel*E	15 (83.3%)	13 (81.25%)	N.S.	7 (70%)	10 (90.9%)	10 (100%)	N.S.
	*esp*	0	1 (6.25%)	N.S.	0	1 (9.1%)	0	N.S.
	*agg*	0	1 (6.25%)	N.S.	0	1 (9.1%)	0	N.S.

*Abbreviations*: *TE* tetracycline, *E* erythromycin, *CN* gentamicin, *P* penicillin, *VAN* vancomycin, *tet*(M) tetracycline resistance gene, *erm*(B) macrolide resistance gene, *aac (6′)-Ie-aph (2″)-Ia* aminoglycoside resistance gene, *pbp5* penicillin resistance gene, *cylA* cytolisin A gene, g*elE* gelatinase E gene, *esp* enterococcal surface protein gene, *agg* aggregation substance gene, *N.S.* non-significant

Resistance to tetracycline, erythromycin, gentamicin and penicillin was observed in isolates recovered from broilers reared in both farming systems. In contrast, vancomycin-resistant enterococci were not found in the present study. When comparing each farming system, 14 (77.7%) indoor broiler isolates and 14 (87.5%) free-range broiler isolates were resistant to tetracycline, the antibiotic to which the enterococci studied presented the highest resistance frequencies. Resistance to erythromycin was found in 10 (55.6%) indoor and in six (37.5%) free-range broiler isolates. Gentamicin resistance was observed in nine (50%) of the isolates from indoor broilers versus seven (43.8%) from free-range broilers. Lastly, resistance to penicillin was lower, as six (33.3%) isolates from indoor broilers and only one (6.25%) isolate of free-range broilers were found to be resistant.

The frequencies of resistance to the antibiotics tested were not associated with the type of farming system in which the broilers were reared in. On the other hand, resistance to penicillin was associated with the genomic pattern similarity clusters (*p* = 0.002), with a higher frequency of resistance observed in *E. faecium* cluster A isolates (60%) when compared with the isolates of cluster B (0%) or with *E. faecalis* cluster isolates (10%).

The distribution of the *tet*(M) gene among the enterococci was similar between the two groups, including nine (50%) indoor and nine (56.25%) free-range isolates. All of the *tet(*M) gene positive isolates were phenotypically resistant to tetracycline. The *erm*(B) gene that confers resistance to macrolides was found in eight (44.4%) of the conventional broiler enterococci and in four (25%) from non-conventional broilers. More than half (56.25%) of erythromycin resistant isolates harbored the *erm*(B) gene. The aminoglycoside resistance gene *aac (6′)-Ie-aph (2″)-Ia* was found in five (27.7%) of the enterococci isolated from indoor broilers and in only one (6.25%) isolate from free-range broilers. Five out of six isolates harboring this resistance gene were phenotypically resistant to gentamicin, regardless of the farming system. On the other hand, *aac (6′)-Ie-aph (2″)-Ia* gene was not observed in 11 gentamicin resistant isolates, suggesting that they probably harbor another gentamicin-resistance determinant, not screened for in the present study. The β-lactam resistance gene *pbp5* was detected in six (33.3%) isolates recovered from indoor broilers and in one (6.25%) from free-range broilers. All of the penicillin-resistant isolates were positive for the *pbp5* gene.

Although no associations were found regarding the resistance genes tested and either of the production systems, the association between the presence of *pbp5* gene and RAPD patterns was statistically significant (*p* = 0.002), with a higher frequency of *E. faecium* cluster A isolates harboring that resistance gene (60%) when compared with cluster B isolates B (0%) or with *E. faecalis* isolates (10%).

Overall, approximately one fourth (23.5%) of the enterococci isolates studied, six indoor broiler isolates and two free-range isolates, were found to be resistant to three or more of the antimicrobial compounds tested. The most frequent combined resistance pattern was TE/E/CN (5/8 isolates), followed by TE/E/P (2/8 isolates) and by TE/E/CN/P (1/8 isolates). Genetically, more than half (55.5%) of the isolates from the indoor broilers harbored at least two antibiotic resistance genes, contrasting with the scenario found in the isolates from outdoor broilers, where only 3 isolates (18.75%) had two resistance genes.

### Virulence phenotype analysis

Results regarding the virulence factors studied can be found in Table 1. Six (33.3%) indoor broilers isolates and 3 (18.75%) free-range broilers isolates presented hemolytic activity, while 8 (44.4%) indoor broilers isolates and 9 (56.25%) fee-range broiler isolates presented gelatinase activity. None of the enterococcal isolates evaluated in the present study presented hemolytic and gelatinase activity simultaneously. None of the virulence phenotypes were found to be associated with any of the farming systems, yet both hemolytic (*p* = 0.004) and gelatinase (*p* = 0.003) activity were found to be associated with RAPD patterns.

### Virulence genes

Results regarding the detection of virulence genes in the isolates studied are presented in Table 1. None of the broiler enterococci isolates harbored the cytolysin coding gene *cyl*A. Contrasting, *gel*E was found in the majority of the isolates, including 15 (83.3%) indoor broiler enterococci and 13 (81.25%) free-range enterococci. The presence of *esp* and *agg* was rare, as only one *E. faecium* free-range broiler isolate harbored both these genes, along with the *gel*E gene. No statistical associations between the presence of virulence related genes and farming systems or RAPD patterns were found.

## Discussion

Dissemination of antibiotic resistant bacteria via food-producing animals and food chains has been regarded as a public health concern during the past years, though a clear demonstration that food-producing animals may represent a pool of resistant bacteria and resistance determinants with an actual impact on human health can be a demanding exercise [18]. Even so, enterococci, more specifically *E. faecium* and *E. faecalis*, have emerged as important multidrug resistant healthcare associated bacteria, in some extent due to the extensive use of antibiotics, not only in human but also in veterinary medicine [19].

With the present work we sought to compare the differences in AMR profiles and virulence genes found in enterococci isolated from fecal samples of healthy broilers raised in Portugal, under conventional indoor or free-range rearing conditions. More than half of the isolates were identified as *E. faecium* and about a third as *E. faecalis*, which is in agreement with other researchers, who reported similar results in Australia [17] and Canada [20]. After RAPD PCR and clustering analysis, results indicated that the clusters identified included isolates from indoor and free range broilers, revealing that there was not an association between genomic profile and farming system. Even though the animals from which the fecal samples were collected had been raised in different housing conditions, although unlikely, if they were obtained from the same hatchery there could be an early colonization with similar enterococci before being transferred to different broiler farms. In a previous work, the horizontal dissemination of *E. faecalis* within the hatcher was proposed to occur via oral route and by cloacal “drinking” between chicks, since these animals can initially be exposed to these bacteria by contact with contaminated eggshells or embryos [21].

Antimicrobial resistance is one of the most notorious features of enterococci. Overall, there were no differences between farming systems regarding phenotypic resistance to any of the antibiotics tested. Tetracycline resistance was observed in the majority of isolates from both farming systems, which is in accordance with recent studies that also reported high incidences of tetracycline resistance in poultry enterococci from different countries [14, 17, 22, 23]. The *tet*(M) gene was detected in 52.9% of the enterococci and more than half of the tetracycline resistant isolates exhibited this gene, suggesting the existence of other tetracycline resistance genes among the bacteria studied.

Phenotypical resistance to gentamicin and erythromycin was observed in about half of all isolates, independently of the farming system. These results are in accordance with a previous study performed in Australia in which resistance to gentamicin and erythromycin was also detected in approximately half of the enterococcal isolates, from both indoor and free-range broilers and free-range layer hens [17].

In the present study, the incidence of penicillin resistance was quite lower in free-range broiler enterococci, though no statistical association was found. This antimicrobial resistance seemed to be associated with the presence of the *pbp5* gene, since all penicillin-resistant isolates harbored this β-lactam resistance gene. Additionally, the majority of the penicillin resistant isolates belonged to the *E. faecium* cluster A, comprising isolates recovered from conventional and free-range broilers.

Remarkably, no vancomycin-resistant enterococci (VRE) were detected in the studied samples. During the last decades, detection of VRE in poultry or poultry meat has been reported in different European, American and Asian countries [24–30]. Portugal is not an exception, since vancomycin resistance has been detected not only in enterococci from poultry fecal samples [31], but also in broiler feed [32] and even in wastewater and sludge samples from poultry slaughterhouses [33].

Animal production systems based on better housing and animal welfare conditions are pointed out as means to reduce the amounts antibiotics used by conventional systems thus, hypothetically, diminishing the number of AMR bacteria arising from food-producing animals. In accordance, some authors have found that it is more likely to find multidrug resistant enterococci in conventional chicken meat than in “organic” chicken meat, and that non-conventional farming practices contribute for a decreased dissemination of antibiotic resistance [16]. Our results, despite the possible effect of the reduced sample size, do not support this view, since no statistically significant differences in antibiotic resistance were found when comparing the two farming systems, but only with the RAPD clusters. Though the free-ranging systems should provide better rearing environments those are not “antibiotic-free” farms. In a recent study focusing on the factors associated with the use antibiotics in French free-range broiler flocks, in the vast majority of cases antibiotics were used with a therapeutic purpose, to control abnormal mortality rates and digestive disorders [34].

About one fourth (26.47%) of all enterococcal isolates studied exhibited hemolytic activity, and half (50%) of the isolates demonstrated a gelatinase phenotype, though none of the enterococcal isolates tested presented both phenotypes. Regarding the virulence determinants investigated, none of the enterococci harbored the *cyl*A gene. The presence of a hemolytic phenotype in *cyl*A gene negative enterococcal isolates, or in the absence of other *cyl* operon genes such as *cyl*B and *cyl*C, has already been reported, suggesting that hemolytic activity might be associated with other genetic determinants [20]. Additional studies on this matter are essential, since it appears that there is no direct correlation between β-hemolysis and the detection of *cyl* operon genes in *Enterococcus* isolates from poultry [35]. On the other hand, *gel*E was the most frequent virulence gene, as it was found in enterococcal isolates from both farming systems at similar rates. All isolates expressing a gelatinase phenotype harbored *gel*E gene, except for one *E. faecium* isolate. Similar results have been reported in a recent publication, where 36.4% of gelatinase producing *E. faecium* human clinical isolates did not have a corresponding *gel*E positive genotype [36]. In contrast, 12 *gel*E positive isolates did not produce gelatinase, supporting previous results highlighting that this gene is frequently silent in enterococcal isolates [36, 37]. Enterococcal gelatinase activity may play an important role on the permeability of enterocytes [38, 39], allowing enterococci to gain access to the deeper layers of the intestine, hence having paramount importance in human food-borne infections. The prevalence of the *esp* and *agg* genes seems to be uncommon among poultry enterococci since only one of the studied isolates from the free-range system harbored these genes simultaneously. These determinants, along with *gel*E, are associated with the capability of enterococci to form biofilms, a community of microorganisms which can adhere strongly to abiotic and biotic surfaces [40]. One study has shown the ability of human and animal enterococci to produce biofilms, revealing that animal enterococci had a lower capability for biofilm formation [41]. Nevertheless, about half of all enterococcal isolates from indoor and free-range broilers were capable of producing biofilms, already reported in previous study [42]. Further research is required to assess the actual contribution of *gel*E, *esp* and *agg* genes in the formation of poultry associated biofilms.

## Conclusions

Despite a similar AMR profile between poultry isolates obtained from farming systems, except for penicillin, the free-range broiler enterococci harbored less resistance genes, though the two farming systems could not be considered statistically different. Results obtained suggest that poultry raised in Portugal, both in conventional indoor or under free-ranging conditions, may act as a reservoir of antibiotic-resistant enterococci. A low incidence of virulence genes was detected among broiler-enterococci, equally distributed between isolates recovered from both farming systems, pointing towards a putative low virulence potential.

## Methods

### Bacterial isolates

The thirty-four enterococci used in this study were isolated from fecal samples obtained from healthy broilers raised in extensive (*n* = 18) and intensive (*n* = 16) farming systems. These isolates belong to a collection of intestinal commensal bacteria from poultry slaughtered for human consumption in two Portuguese slaughterhouses, one of which only slaughtered indoor poultry, characterized by broiler flocks reared in high stocking density (14 to 20 broilers/m2) until 30 to 40 days of age. The other plant exclusively slaughtered broilers from free-range farms, reared at low stocking density in open broiler houses until they reach 81 days of age.

Isolates were identified through their biochemical profile (API 20Strep, BioMérieux, Marcy-l’Etoile, France) as previously described [42].

### DNA extraction

Broiler-isolates were cultured in Brain Heart Infusion (BHI) broth (Scharlau, Barcelona, Spain) at 37 °C for 48 h. DNA extraction was then performed using the guanidium thiocyanate method [43], followed by genotyping and PCR screening for antimicrobial resistance and virulence determinants. Briefly, enterococcal cultures where centrifuged at 1000 g for 15 min, and the supernatant was discarded. The cell pellet was resuspended in 100 μl of a lysozyme (50 mg/ml) TE buffer solution, and incubated for 30 min at 37 °C. After incubation, the bacterial cells were lysed in 500 μl of a guanidium thiocyanate (5 mol/l), EDTA (100 mmol/l) and sarkosyl (0.5% v/v) solution and the cell suspensions were vortexed. Cell lysate was then cooled on ice and 250 μl of cold ammonium acetate (7.5 mol/l) were added and mixed. After 10 min on ice, 500 μl of chloroform and 2-pentanol (24:1) mixture was added and phases carefully mixed. The mixture was transferred into a 1.5 ml clean tube and centrifuged at 25000 g for 10 min. The resulting supernatant was transferred to a new 1.5 ml clean tube and 0.54 volumes of cold 2-propanolol was added and the solution was mixed by inverting the tube for 1 min. The fibrous DNA was precipitated by centrifugation at 6500 g for 20 s and the DNA pellet was washed five times with 70% ethanol. Finally, the DNA pellet was dried under vacuum.

### Microbial diversity

Genomic typing was carried out by PCR-fingerprinting using primers OPC19 (*5′-GTTGCCAGCC-3′*) and (GTG)_5_ [44]. PCR mixture contained 1X reaction buffer, 0.2 mM of each deoxynucleoside triphosphate, 2.5 mM MgCl_2_, 0.5 μM primer, 2 U of Taq DNA polymerase and 100 ng of enterococcal DNA, in a final volume of 25 μl. Amplification was performed in a thermocycler (Biometra, Gottingen, Germany) under the following conditions: initial denaturation step at 95 °C for 5 min, 40 cycles of 1 min at 95 °C, 2 min at 40 °C and 2 min at 72 °C, followed by a final extension step at 72 °C for 10 min. The resulting PCR products were resolved by electrophoresis at 80 V for 2 h 45 min, using an agarose gel in 0.5X TBE buffer (1.2%, w/v). After staining with ethidium bromide, the gels were photographed (Kodak 1D image analysis software, Eastman Kodak Co., New York, NY) before further analysis.

### Antibiotic resistance detection

Enterococcal susceptibility profile to five antimicrobial compounds was evaluated using the disk diffusion method as recommended by the Clinical and Laboratory Standards Institute guidelines [45]. The following compounds were used: gentamicin (CN, 120 μg), penicillin (P, 10 U), erythromycin (E, 15 μg), tetracycline (TE, 30 μg) and vancomycin (VAN, 30 μg). All antibiotics were purchased from Oxoid. Genetic resistance profiling was carried out through PCR-based detection of antibiotic resistance determinants. Previously described primers and conditions for the amplification of resistance genes for macrolides [*erm(B)*], β-lactams [*pbp5*], aminoglycosides *[aac (6′)-Ie-aph (2″)-Ia*] and tetracycline [*tet(M)*] were applied [46]. The reference strain *E. faecalis* CECT 795 (ATCC 29212) was used as quality control.

### Virulence phenotypes analysis

Hemolytic and gelatinase activities were assessed through plate tests [46]. Production of hemolysin was determined by streaking enterococcal cultures (grown overnight in BHI plates) on Columbia agar supplemented with 5% horse blood. Plates were incubated at 37 °C for 72 h under anaerobic conditions, after which they were examined for hemolysis. Presence or absence of clear halos around the colonies were interpreted as β-hemolysis (positive) or γ-hemolysis (negative) activity, respectively.

Gelatinase activity was detected by streaking the BHI enterococcal cultures on 3% gelatin medium. After incubation for 48 h at 37 °C, plates were flooded with a saturated solution of ammonium sulphate and positive strains showed a transparent halo around the colonies.

### Virulence gene detection

Regarding the identification of virulence determinants, all enterococcal isolates studied were screened for the presence of *cyl*A, *gel*E, *esp* and *agg* genes by PCR using the primers and conditions previously described by [46] (Table 2). *E. faecalis* MMH594 [47] and *E. faecalis* P1 [48] were included in this study as control strains for the PCR-based screening for virulence determinants.

**Table 2:** Primers used for genetic resistance profiling and virulence gene detection by PCR

Gene	Primers	Product size (bp)
*tet*(M)	5′-ACAGAAAGCTTATTATATAAC-3′ 5′-TGGCGTGTCTATGATGTTCAC-3′	171
*erm*(B)	5′-GAAAAGGTACTCAACCAAATA-3′ 5′-AGTAACGGTACTTAAATTGTTTAC-3	639
*aac(6′)-Ie-aph(2″)-Ia*	5′-CCAAGAGCAATAAGGGCATA-3′ 5′-CACTATCATAACCACTACCG-3′	369
*pbp5*	5′ CATGCGCAATTAATCGG 3′ 5′ CATAGCCTGTCGCAAAAC 3′	444
*cyl*A	5′-TAGCGAGTTATATCGTTCACTGTA-3′ 5′-CTCACCTCTTTGTATTTAAGCATG-3́	1282
*gel*E	5′-ACCCCGTATCATTGGTTT-3′ 5′-ACGCATTGCTTTTCCATC-3´	419
*esp*	5´-TTGCTAATGCTAGTCCACGACC −3′ 5′-GCGTCAACACTTGCATTGCCGAA-3´	993
*agg*	5´-AAGAAAAAGAAGTAGACCAAC-3′ 5′-AAACGGCAAGACAAGTAAATA-3´	1553

*Abbreviations*: *tet*(M) tetracycline resistance gene, *erm*(B) macrolide resistance gene, *aac (6′)-Ie-aph (2″)-Ia* aminoglycoside resistance gene, *pbp5* penicillin resistance gene, *cyl*A cytolisin A gene, *gel*E gelatinase E gene, *esp* enterococcal surface protein gene, *agg* aggregation substance gene, bp base pairs

### Data analysis

BioNumerics software (version 7.5, Applied Maths, Kortrijk, Belgium) was used to register genomic patterns, normalize densitometric traces, calculate the Pearson product-moment correlation coefficient, and to perform cluster analysis by the unweighted pair group method with arithmetic mean algorithm (UPGMA). Reproducibility of all above-mentioned methods was assessed by analyzing a random sample of 10% duplicates.

Associations between antimicrobial resistance phenotype, antimicrobial resistance genes, virulence phenotype, virulence genes and farming system or RAPD clusters were evaluated using the Fisher exact test. Statistical analysis was performed using SPSS for Windows version 15.0 (SPSS Inc., Chicago, IL). Associations were considered to be significant when *p* values were less than 0.05.

## Data Availability

All data generated or analysed during this study are included in this published article.

## Abbreviations

*aac (6′)-Ie-aph (2″)-Ia*: Aminoglycoside resistance gene
*agg*: Aggregation substance gene
AMR: Antimicrobial resistance
bp: Base pairs
*cyl*A: Cytolisin A gene
CN: Gentamicin
E: Erythromycin
*erm*(B): Macrolide resistance gene
*esp*: Enterococcal surface protein gene
*gel*E: Gelatinase E gene
P: Penicillin
*pbp5*: Penicillin resistance gene
TE: Tetracycline
*tet*(M): Tetracycline resistance gene
VAN: Vancomycin
VRE: Vancomycin-resistant enterococci
